# Supplementary material for: Variation in COVID-19 booster uptake in England: An ecological study
Source: PLoS One. 2022 Jun 29;17(6):e0270624. doi: 10.1371/journal.pone.0270624 (PMC9242486; doi:10.1371/journal.pone.0270624)
Supplement: S1 File — Guide to the files contained within the zip files for codes, data, and outputs, how to run the codes, and short simulations to illustrate second stage modelling. (PDF) [file pone.0270624.s001.pdf]

Guide

Files used to run the models are archived in S2_File.zip ("Codes"), S3_File.zip ("Data1"), S4_File.zip ("Data2"), S5_File.zip ("Data3"), S6_File.zip ("Output"). All should be extracted and moved to the working directory. This is called "use" in the “models” file.

Once all these files are in "use", the codes can be run.

Separately, and not needed for running the codes, S7_File.pdf is a summary output for model $\mathcal{D}$ applied to the Third Injection. S1_Appendix.pdf is a brief mathematical outline of the method of fitting GAM models and optimising the choice of smoothing parameters.

Code files

"models": The main file, from which all others are called, except for "simutest1"
"getdata": called from "models" to load the data and define variables.
"density.txt": used to make "allmat2.txt"
"make_alldatr": A utility file to make "alldatr.txt"
“pcvxf”: uses postcodes for vaccination sites to get numbers of sites in each UTLA, per population
"simutest1": simulations to illustrate the methods in 2nd stage modelling. see further below.

Data1 files

"alldatr.txt": load for "alldatr", the matrix of incidence rates for 6789 MSOA and 96 weeks.
"allmat2.txt": load for allmat2, the matrix of MSOA densities and populations
"allvariableslsoawdeciles.csv": Data file from the AHAH dataset at LSOA level
"EDW.txt": load for EDW, weighted average distances in MSOA to Emergency Departments.
"ethnic2011.csv”: ethnicity data from 2011 census
"fluvac1.csv": Flu vaccination rates
"GPR.txt": load for GP registrations at MSOA level
"GPW.txt": load for GPW, weighted average distances in MSOA to GP surgery
"house1.csv": Census data on multi-occupation housing
"IMDA.txt": load for IMDA, the weighted average MSOA level IMD
"IMDE.txt": load for IMDE, the weighted average MSOA level Education deprivation domain
"IMDH.txt": load for IMDH, the weighted average MSOA level Health deprivation domain
"indusexage1.csv": Employment by industrial sectors
"lookup_table.csv": lookup between MSOA, LTLA, UTLA, Region
"lsoapop_2019.csv": LSOA level populations
"malepop1.csv": male populations
"mariano.wav": sound file for beep
"pcgp": postcodes for GP-led vaccination sites
"pchh": postcodes for hospital hub vaccination sites
"pcphm": postcodes for pharmacy vaccination sites
"pcvxctr": postcodes for vaccination centres
"phallo20-21.csv": Ring-fenced Public Health allocations 2020-21
"phallo21-22.csv": Ring-fenced Public Health allocations 2021-22
"PHMW.txt": load for PHMW, weighted average distances in MSOA to Pharmacy
"pop2019.csv": age-specific populations
"prison1.csv": Census data on communal and prison populations
"vacc_2022-01-04.csv": cumulative vaccinations (1st dose, 2nd dose, 3rd Injection)

Data2 files

"gp-reg-pat-prac-lsoa-all.csv": GP registrations at LSOA level
"imd2019lsoa.csv": Index of Multiple Deprivation and domains, at LSOA level

Data3 files
"msoa_2022-01-04.csv": incidence rates for each week and MSOA (to use with "alldatr.txt")
"Output_lookup.csv": lookup for OA,LSOA,MSOA,LTLA, Region

Output files
"cf.txt": load for random effects coefficients from 1st stage models, used in 2nd stage modelling

"GPP.txt": load for number of GP-led vaccination sites in each UTLA, per population
"HHP.txt": load for number of hospital hub vaccination sites in each UTLA, per population
"PHMP.txt": load for number of pharmacy vaccination sites in each UTLA, per population
"VXCP.txt": load for number of vaccination centres in each UTLA, per population

"SCSP4xre.txt": load for saved smoothing parameters from mod4x1re, mod4x2re, and mod4x3re, from which the fitted model can be recovered without full refitting.
"SCSP6xre.txt": likewise for mod6x1re etc
"SCSP8xre.txt": likewise for mod8x1re etc
"SCSP10xre.txt": likewise for mod10x1re etc

"UTLA.qgs": Qgis file for Fig 4

Simulation
The code file "simutest1" has simulations to illustrate the techniques in 2nd stage modelling

#1: generate 4 uniform covariates, apply 4 functions, and sum the results to form a linear predictor from which scaled binomial data is generated. Model it as a quasibinomial gam to recover the scale and the functions.

#2: adjust the linear predictor using random coefficients applied to a factor variable. Generate scaled binomial data and model it including a random effects smoother, to recover the scale, functions, and estimated random effects.

#3: as in #2, but now the coefficients applied to the factor are themselves generated as a univariate linear model with error term. Model as in #2 to estimate random effects, and model them to recover the univariate coefficient.

#4: coefficients applied to the factor as in #3 are generated as a univariate linear model with error term plus a 2nd factor term with its 2nd level coefficients. Estimate the random effects, and apply a mixed model in stan_lmer to recover the univariate coefficient and the 2nd level random effects.

#5: as in #4, but simulate the random effects using the estimates and their corrected covariance matrix, to give repeated simulations to which stan_lmer is applied.
