# Supplementary material for: Variation in COVID-19 booster uptake in England: An ecological study
Source: PLoS One. 2022 Jun 29;17(6):e0270624. doi: 10.1371/journal.pone.0270624 (PMC9242486; doi:10.1371/journal.pone.0270624)
Supplement: S7 File — Summary of model D applied to the Third Injection. (PDF) [file pone.0270624.s007.pdf]

Summary of model $\mathcal{D}$ applied to the Third Injection

summary(mod10x3re)
Family: quasibinomial   Link function: logit

Formula:
vmat3 ~ s(utla, bs = "re") + s(zCSUM, bs = "cr", k = 7) + s(zIMD, bs = "cr", k = 7) + s(LPROP04, bs = "cr", k = 7) + s(LPROP514, bs = "cr", k = 7) + s(LPROP1524, bs = "cr", k = 7) + s(LPROP2544, bs = "cr", k = 7) + s(LPROP4564, bs = "cr", k = 7) + s(LPROPM, bs = "cr", k = 7) + s(LWhIriGyp, bs = "cr", k = 7) + s(LOthWh, bs = "cr", k = 7) + s(LSAsian, bs = "cr", k = 7) + s(LAsian, bs = "cr", k = 7) + s(LAfrCarOth, bs = "cr", k = 7) + s(LArab, bs = "cr", k = 7) + s(LAgEnWat, bs = "cr", k = 7) + s(LConstr, bs = "cr", k = 7) + s(LWholRetl, bs = "cr", k = 7) + s(LTport, bs = "cr", k = 7) + s(LFinIns, bs = "cr", k = 7) + s(LProfSci, bs = "cr", k = 7) + s(LAdmin, bs = "cr", k = 7) + s(LPubAdmin, bs = "cr", k = 7) + s(LEduc, bs = "cr", k = 7) + s(LHlthSw, bs = "cr", k = 7) + s(LOthInd, bs = "cr", k = 7) + s(LCommun, bs = "cr", k = 7) + s(LHous, bs = "cr", k = 7) + s(LEDW, bs = "cr", k = 7) + s(LPHMW, bs = "cr", k = 7) + s(GPR, bs = "cr", k = 7) + s(IMDH, bs = "cr", k = 7) + s(IMDE, bs = "cr", k = 7) - 1

Approximate significance of smooth terms:

term	edf	Ref.df	F	p-value	
s(utla)	147.753	149.000	202.705	<2e-16	***
s(zCSUM)	1.001	1.003	28.159	1.12e-07	***
s(zIMD)	3.691	4.395	67.178	<2e-16	***
s(LPROP04)	2.307	3.004	8.861	7.32e-06	***
s(LPROP514)	5.096	5.594	81.391	<2e-16	***
s(LPROP1524)	3.365	3.945	297.111	<2e-16	***
s(LPROP2544)	4.535	5.221	165.933	<2e-16	***
s(LPROP4564)	3.334	4.052	12.157	6.63e-10	***
s(LPROPM)	3.718	4.355	19.589	<2e-16	***
s(LWhIriGyp)	4.748	5.364	8.048	5.19e-08	***
s(LOthWh)	4.427	5.000	180.483	<2e-16	***
s(LSAsian)	3.500	3.952	94.571	<2e-16	***
s(LAsian)	2.939	3.519	7.350	2.91e-05	***
s(LAfrCarOth)	3.233	3.696	133.969	<2e-16	***
s(LArab)	2.754	3.248	8.089	1.39e-05	***
s(LAgEnWat)	2.831	3.487	6.437	0.000114	***
s(LConstr)	3.693	4.464	15.106	4.49e-13	***
s(LWholRetl)	4.847	5.438	9.075	5.63e-09	***
s(LTport)	3.630	4.338	5.671	0.000128	***
s(LFinIns)	3.932	4.614	11.046	1.02e-09	***
s(LProfSci)	3.444	4.208	9.176	1.68e-07	***
s(LAdmin)	3.617	4.365	8.157	7.79e-07	***
s(LPubAdmin)	2.838	3.460	12.190	1.20e-08	***
s(LEduc)	5.201	5.700	7.324	1.14e-07	***
s(LHlthSw)	2.020	2.597	31.592	<2e-16	***
s(LOthInd)	4.877	5.432	8.929	1.40e-08	***
s(LCommun)	4.887	5.407	14.426	7.35e-15	***
s(LHous)	3.490	4.130	7.642	3.09e-06	***
s(LEDW)	3.407	4.110	5.755	0.000111	***
s(LPHMW)	2.982	3.525	3.967	0.004389	**
s(GPR)	4.483	4.973	59.128	<2e-16	***
s(IMDH)	3.502	4.378	8.256	4.37e-07	***
s(IMDE)	5.543	5.880	8.961	1.06e-08	***

Signif. codes: 0 ‘***’ 0.001 ‘**’ 0.01 ‘*’ 0.05 ‘.’ 0.1 ‘ ’ 1

R-sq.(adj) = 0.972 Deviance explained = 97.2%
-ML = -16223 Scale est. = 20.689 n = 6789
