# Supplementary material for: Variation in COVID-19 booster uptake in England: An ecological study
Source: PLoS One. 2022 Jun 29;17(6):e0270624. doi: 10.1371/journal.pone.0270624 (PMC9242486; doi:10.1371/journal.pone.0270624)
Supplement: S1 Appendix — Brief outline of how GAM models are fitted, smoothing parameter optimisation by “Marginal Likelihood”, and the standard errors of fitted coefficients. (PDF) [file pone.0270624.s008.pdf]

Appendix

Other supplementary material including a file guide, simulations to illustrate first and second stage modelling, and code files can be read (and run) without reference to this appendix, which outlines how GAM models are fitted, “Marginal Likelihood”, and the standard errors of fitted coefficients.

Fitting a GAM involves maximising a penalised log likelihood (PLL), in which a term expressing the complexity of the model is subtracted from the log likelihood. This has the form

$$PLL = \Sigma_r LL_r - 1/(2*\varphi)*\boldsymbol{\beta}'*\mathbf{S}*\boldsymbol{\beta}$$

where LL_r is the log likelihood of y_r, arising from the presumed distribution of y_r from a specified exponential family, φ is the dispersion, $\boldsymbol{\beta}$ is the vector of fitted model coefficients and $\boldsymbol{\beta}'$ is its transpose, and $\mathbf{S}$ is the penalty matrix, generally a direct sum of matrices $\mathbf{S_j}$ corresponding to the smooth terms in the model. When the smooths are univariate, as in this paper, each $\mathbf{S}_j$ has the form $sp_j*\mathbf{S}_{j0}$ where sp_j is an unknown smoothing parameter, to be optimised during fitting, and $\mathbf{S}_{j0}$ is determined by the structure of the spline term, before fitting. In our case, the penalty matrix for the random intercept term is $\mathbf{S}_1 = sp_1*I_{149}$ where I_{149} is the square diagonal matrix whose 149 diagonal entries are all 1. In this formulation, sp_1 / φ is the inverse of the variance of the random intercept. In general, lower values of the smoothing parameters give lower penalties, allowing the fitted values to come closer to the observed y_r , whilst higher smoothing parameters cause greater smoothing of the data away from the observed values in exchange for simplifying the model. The compromise between these two conflicting goals is determined by the choice of smoothing parameters.

One of many sophisticated techniques for choosing smoothing parameters is “Marginal Likelihood”,[1] in which the penalised likelihood exp(PLL) is reinterpreted as a posterior likelihood, where the likelihood is given by the model and the prior is a function of the unknown coefficients $\boldsymbol{\beta}$ along with φ and $\mathbf{S}$, a multiple of $\exp(-1/(2*\varphi)*\boldsymbol{\beta}'*\mathbf{S}*\boldsymbol{\beta})$. A Laplace approximation to this posterior is integrated (marginalised) over those $\boldsymbol{\beta}$ which are orthogonal to the kernel of $\mathbf{S}$, i.e. over the $\boldsymbol{\beta}$ orthogonal to those which are unpenalised and do not contribute to the prior. This integral is a function of the smoothing parameters alone (as the S_{j0} are fixed by the choice of splines, and φ is estimated separately). The optimised smoothing parameters are those which maximise the marginalised posterior likelihood. The ML value as reported by “mgcv” is the negative log of this maximal marginalised posterior likelihood (with a further adjustment when using a “quasi” family).

Another output reported for a fitted model M is the “proportion of Deviance explained”. This is (Deviance(M_0) – Deviance(M))/Deviance(M_0) where M_0 is the null model $g(\mu_r)$ = constant, fitted with the same family as M. For linear models this is R^2, the proportion of variance explained.

The fitted output includes the fitted model coefficients b and their Bayesian posterior covariance matrix V_c which includes correction for smoothing parameter uncertainty (an option when using marginal likelihood). The posterior distribution of the coefficients $\boldsymbol{\beta}$ is approximately normal with mean b and covariance V_c , an approximation which holds for large samples. Thus each fitted value β_k also has an approximate standard error obtained as the square root of the kth diagonal term of V_c , which can be used to obtain credible intervals. If $\boldsymbol{\beta}_{idx}$ denotes a subset of the coefficients as indexed by idx, the posterior covariance of $\boldsymbol{\beta}_{idx}$ is V_c [idx,idx].

Although the penalised likelihood has been given a Bayesian interpretation, this is not a Bayesian model. Samples are not being drawn from the full posterior, and the fitting depends on a particular method of choosing the smoothing parameters followed by maximising the penalised log likelihood.

---

1 Wood, S.N., N. Pya and B. Saefken (2016), Smoothing parameter and model selection for general smooth models. Journal of the American Statistical Association 111, 1548-1575 https://doi.org/10.1080/01621459.2016.1180986
